# Preclinical evaluation of the versius surgical system, a new robot-assisted surgical device for use in minimal access general and colorectal procedures

**DOI:** 10.1007/s00464-020-07622-4

**Published:** 2020-05-13

**Authors:** Jonathan Morton, Richard H. Hardwick, Henry S. Tilney, A. Mark Gudgeon, Asif Jah, Lewis Stevens, Slawomir Marecik, Mark Slack

**Affiliations:** 1 Department of Gastrointestinal Surgery, Cambridge University Hospitals NHS Foundation Trust, Addenbrooke’s Hospital, Cambridge, UK; 2 Department of Surgery, Frimley Health NHS Foundation Trust, Frimley Park Hospital, Surrey, UK; 3 Cambridge Hepato-Pancreato-Biliary and Transplant Centre, Cambridge University Hospitals NHS Foundation Trust, Addenbrooke’s Hospital, Cambridge, UK; 4 Department of Molecular Oncology, Barts Cancer Institute, Queen Mary University London, London, UK; 5 Division of Colon and Rectal Surgery, Advocate Lutheran General Hospital and University of Illinois at Chicago, Illinois, USA; 6 CMR Surgical Ltd, 1 Evolution Business Park, Milton Road, Cambridge, CB24 9NG UK

**Keywords:** Minimally invasive surgical procedures, Robotic surgical procedures, General surgery, Colorectal surgery

## Abstract

**Objective:**

To evaluate the utility of a new robot-assisted surgical system (the Versius Surgical System, CMR Surgical, Cambridge, UK) for use in minimal access general and colorectal surgery, in a preclinical setting.

**Summary background data:**

Robot-assisted laparoscopy has been developed to overcome some of the important limitations of conventional laparoscopy. The new system is designed to assist surgeons in performing minimal access surgery and overcome some of the challenges associated with currently available surgical robots.

**Methods:**

Cadaveric sessions were conducted to evaluate the ability of the system to provide adequate surgical access and reach required to complete a range of general and colorectal procedures. Port and bedside unit positions were recorded, and surgical access and reach were evaluated by the lead surgeon using a visual analogue scale. A live animal (porcine) model was used to assess the surgical device’s safety in performing cholecystectomy or small bowel enterotomy.

**Results:**

Nine types of procedure were performed in cadavers by nine lead surgeons; 35/38 procedures were completed successfully. The positioning of ports and bedside units reflected the lead surgeons’ preferred laparoscopic set-up and enabled good surgical access and reach. Cholecystectomy (*n* = 6) and small bowel enterotomy (*n* = 5) procedures performed in pigs were all completed successfully by two surgeons. There were no device-related intra-operative complications.

**Conclusions:**

This preclinical study of a new robot-assisted surgical system for minimal access general and colorectal surgery demonstrated the safety and effectiveness of the system in cadaver and porcine models. Further studies are required to assess its clinical utility.

**Electronic supplementary material:**

The online version of this article (10.1007/s00464-020-07622-4) contains supplementary material, which is available to authorized users.

Minimal access surgery (MAS) in general and colorectal specialties was first applied in laparoscopic cholecystectomy over three decades ago, and is now well supported by evidence confirming its efficacy and safety [1–3]. Advantages of MAS over open surgery include lower blood loss, reduced incidence of post-operative adhesions, fewer wound complications, reduced post-operative pain, earlier recovery, shortened hospital stay and improved cosmesis [3–5]. However, competency in MAS, particularly within the confines of the pelvis, is generally associated with a long learning curve; anatomical challenges expose the limitations of conventional laparoscopy, such as restricted range of movement and two-dimensional vision, which make accurate dissection and suturing difficult [4, 6, 7].

Robot-assisted laparoscopy has made progress in overcoming these challenges by providing an ergonomic operating position, a stable magnified three-dimensional view and articulated or wristed instruments allowing for precise tissue dissection and suturing. These improvements have resulted in further reductions in blood loss and hospital stay [2, 8]. Robotic assistance also eases the execution of technically challenging tasks within confined spaces, which may increase the accessibility of MAS to surgeons [2, 3, 9]. Therefore, robotic surgery could extend the feasibility of, and widen the application of, MAS to more complicated and advanced general and colorectal procedures [9].

The Versius Surgical System is a novel tele-operated robotic surgical system (Supplementary Fig. 1) designed to assist surgeons in performing MAS and overcome some of the challenges associated with currently available surgical robots [10, 11]. The device was developed to improve surgeon experience, with the user and patient central to the design. The surgeon interacts with the system through the hand controllers with feedback via the head-up display (HUD), which delivers three-dimensional, high-definition video from the endoscopic camera together with a display overlay showing active instruments, system warnings and system function. The bedside team follows the surgery on a two-dimensional, high-definition version of the endoscope feed via an auxiliary display. They are able to access controls on the visualisation bedside unit (BSU), and on up to four instrument BSUs [12].

Throughout the development of the system, end-user feedback was used to refine the design to ensure it met user needs. The robot mimics the articulation of the human arm, and the wristed instrument tip provides seven degrees of freedom inside the patient, allowing a broader range of surgical access compared with standard laparoscopic surgery. The system’s modular design increases its potential for flexible use, as the BSUs are small enough to be used in a standard operating room (OR) and can easily be moved within a single OR between ORs. The ‘game controller’ handgrip was based on extensive ergonomic research and further developed with surgeon input. Finally, the open console, which is designed for surgeons to sit or stand, encourages better visual and verbal communication with improved situational awareness between the surgeon and the wider operative teams [12].

The operational safety and ease of use of the system were validated in human cadaver studies [13]. The next step in assessing its suitability for use in general and colorectal surgery is preclinical evaluation, as per the IDEAL-D framework and recommendations for surgical innovation [14, 15]. The preclinical studies described herein have two aims: (1) to evaluate the ability of the system to provide adequate surgical access and the reach required to complete a range of general and colorectal procedures using cadavers and (2) to assess the ability to perform cholecystectomy or small bowel enterotomy safely and effectively in a live animal (porcine) model. The latter allows evaluation of the impact of live tissue manipulation in terms of intra-operative bleeding, tissue injury and recovery.

## Methods

### Study design

The human cadaver studies were conducted at the Evelyn Cambridge Surgical Training Centre, UK, and at the AdventHealth Nicholson Centre, USA, between 24th July 2018 and 22nd August 2019. All cadavers were donated with consent. The live animal porcine study was performed at Covance CRS Ltd (formerly Envigo Ltd), Huntingdon, UK between 16th and 22nd October 2018. All procedures were performed in a replicated OR in a manner reflecting how they would be performed in a true clinical setting. The porcine study was conducted in accordance with current, internationally recognised Good Laboratory Practice (GLP) Standards and the UK Animals (Scientific Procedures) Act 1986, Amendment Regulations 2012, and was designed to align with the principles of the 3Rs (replacement, reduction and refinement).

### Surgical team

Procedures were performed by a lead surgeon supported by a surgical team. Lead surgeons included Jonathan Morton, Richard H. Hardwick, Henry S. Tilney, A. Mark Gudgeon, Asif Jah, Slawomir Marecik, Ashish Pradhan, Carlos Vaz, Roger Motson, James Wheeler and Salamone Di Saverio. The lead surgeon performed the surgical steps for the procedure and evaluated the system in line with the objectives of the specific study. The assistant surgeon carried out any additional manual tasks, such as suction or retraction, as instructed by the lead surgeon. Additional personnel present recorded port and BSU placements along with outcomes.

The nine lead surgeons who performed the procedures in cadavers were accredited, practising, high-volume general or colorectal consultant surgeons, as defined by > 50 cases/annum for the procedures performed. The two lead surgeons performing procedures in pigs were also practising consultant surgeons who were GLP trained and certified and possessed UK Home Office licences. All users were trained to use the robot and had experience of performing procedures on the system in prior studies. During the procedures described here, a professional CMR Surgical education team provided expert advice at the console.

### Cadaver studies

A variety of general and colorectal procedures were performed in fresh frozen cadavers or cadaver specimens (torso to mid femur) which had not undergone previous surgery. Cadavers were selected to represent a range of body mass indices (BMIs) to reflect the wide range in size and shape of human anatomy expected to be encountered in the clinical setting.

The lead surgeon determined the port and BSU positions, based on their established, standard technique of performing the same procedure, either by conventional laparoscopic means or robotically using another system. Instrument and accessory ports were inserted either following insufflation using a Veress needle, or using the open Hasson technique. Safe entry and establishment of the pneumoperitoneum were performed using standard surgical techniques.

Port and BSU positions were recorded using a 20 cm grid (covering 320 cm × 320 cm) laid out on the OR floor (Supplementary Fig. 2); BSU positions in relation to anatomical landmarks on the cadaver were also recorded. Port and BSU positions were iteratively altered from one procedure to the next in response to difficulties in surgical access and reach such as inability to reach surgical site, instruments too close to surgical site or arms clashing due to port positioning. Positions were deemed suitable if good access to the surgical site(s) was achieved without arm clashing and there was minimal need to reposition the BSUs. Surgical access and reach for a subset of procedures were evaluated by the lead surgeon using a visual analogue scale (VAS). The precise surgical steps conducted to make the assessment that the procedures could be fully completed were recorded, as well as instruments used (including any manual laparoscopic instruments) and endoscope angle.

### Porcine study

Large White Hybrid domestic female pigs aged 18–20 weeks and weighing 36.5–44.0 kg (mean weight = 41.8 kg) underwent either cholecystectomy or small bowel enterotomy (one procedure per pig). Prior to the procedure, and in accordance with GLP in animal studies, each pig was sedated before transfer to the OR, where the animal was placed under general anaesthesia and intubated. Procedures in pigs were performed by Jonathan Morton and Mark Slack.

During the procedure, intra-operative blood loss was estimated and intra-operative adverse events were recorded. Pigs were divided into two groups: non-recovery and recovery. Non-recovery pigs were euthanised without recovery from anaesthesia with pentobarbitone. Successful and safe procedure completion was confirmed in non-recovery pigs before the procedure was attempted in recovery pigs. In recovery pigs, wounds were closed, anaesthesia was discontinued and animals were observed for signs of ill health or changes in behaviour and/or activity. Post-operative analgesia, antibiotic treatment and other treatments as appropriate were administered by a veterinary surgeon. Recovery pigs were euthanised after 22–29 days and subject to a detailed necropsy, with specific reference to surgical sites and the assessment of successful organ removal and intact anastomosis.

## Ethical approval

All cadaver studies were conducted at The Evelyn Cambridge Surgical Training Centre, Back Lane, Melbourn, Hertfordshire, SG8 6DP, UK. The Evelyn Centre is certified as Health Tissue Authority (HTA) compliant under licence number: 12603. All studies conducted by CMR Surgical at The Evelyn Centre met the required HTA, health and safety, and ethical considerations relating to the use of donated cadaveric tissue in dissection, teaching, research and development. Porcine work was conducted in accordance with the applicable sections of the United Kingdom Animals (Scientific Procedures) Act 1986, Amendment Regulations 2012 (the Act) and in compliance with the requirements of current, internationally recognised Good Laboratory Practice Standards (UK Good Laboratory Practice Regulations; Statutory Instrument 199 No. 3106, as amended by Statutory Instrument 2004 No. 994, OECD Principles of Good Laboratory Practice ENV/MC/CHEM(98)17, and EC Commission Directive 2004/10/EC) and was designed to align with the principles of the 3Rs (replacement, reduction and refinement).

## Results

### Procedure completion in cadavers

**Fig. 1:**
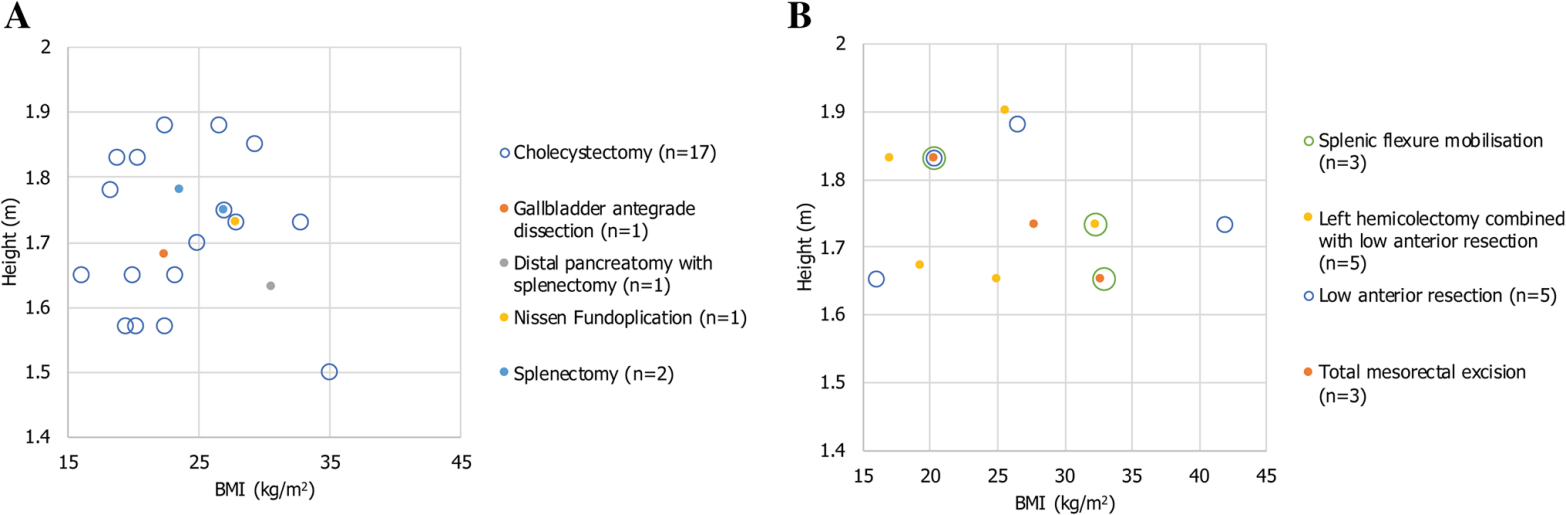
Plots of the range of cadaver BMIs and corresponding heights used for the surgical procedures. **A** BMI and height of cadavers used in general procedures. **B** BMIs and height of cadavers used in colorectal procedures. Note that in some cases multiple procedures were performed on the same cadaver. *BMI* body mass index

The cadavers represented a wide range of BMIs and heights, with BMI ranging from 16.0–42.0 kg/m^2^ (mean BMI = 24.9 kg/m^2^; Fig. 1). In total, nine types of general and colorectal procedures were performed in cadavers across the following anatomical regions: right and left hypochondrium, epigastrium and right and left iliac fossae. These procedures were selected to assess the ability of the system to access specific anatomical regions, and to move across anatomical regions within a single procedure. For procedures executed more than once, there were multiple lead surgeons.

In general, tissue manipulation, dissection and suturing were achieved using the Versius monopolar hook, bipolar Maryland grasper, curved scissors, fenestrated grasper and needle holder. Some procedures also required the use of manual instruments such as graspers, suction/irrigation devices and clip appliers. In total, 38 procedures were performed, of which 35 (92.1%) were successfully completed; two procedures could not be completed due to unsuitable port placement, and one due to the physical condition of the cadaver (Table 1).

**Table 1:** Summary of procedures performed and successful completion in cadavers

Procedure	Number performed	Number successfully completed	Number of lead surgeons	Number of port configurations
Cholecystectomy	17	17	7	6
Gallbladder antegrade dissection	1	1	1	1
Distal pancreatectomy with splenectomy	1	1	1	1
Nissen Fundoplication	1	1	1	1
Splenectomy	2	2	2	2
Splenic flexure mobilisation	3	3	2	3
Left hemicolectomy combined with low anterior resection^a^	5	3	3	5
Low anterior resection^b^	5	4	5	5
Total mesorectal excision^c^	3	3	2	2
Total	38	35 (92.1%)	–	26

^a^One procedure could not be completed due to unsuitable port placement, and in another low anterior resection could not be completed due to faecal impaction of the colon, prohibiting access to the rectum

^b^One procedure could not be completed due to unsuitable port placement

^c^Total mesorectal excision covers a subset of steps required for a full low anterior resection procedure; total mesorectal excision was performed in isolation in three cadavers to further evaluate the robot’s ability to complete this procedure

### Common port and BSU positions in cadavers

**Fig. 2:**
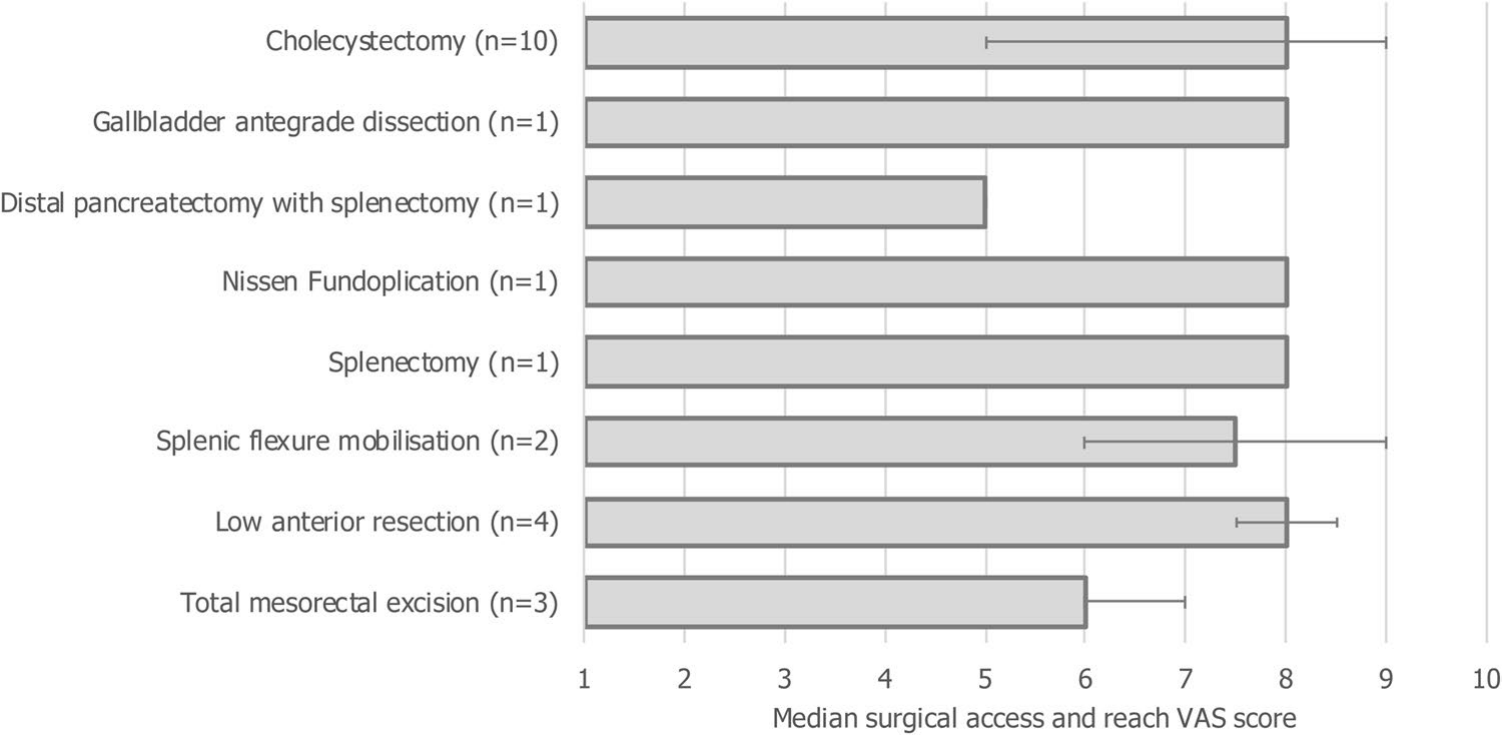
Median surgical access and reach VAS scores for procedures performed in cadavers. Error bars indicate range. VAS, visual analogue scale. VAS scale ranged from 1 (clinically unachievable) to 10 (perfect access). VAS data were unavailable for the procedure type ‘left hemicolectomy combined with low anterior resection’

The port and BSU positions generally reflected the lead surgeon’s standard technique of performing the same procedure laparoscopically, and in some cases robotically using another system. The port and BSU positions enabled good surgical access and reach; for the eight types of procedure in which surgical access and reach were quantified, median VAS was 6 or above in seven of these (Fig. 2). Commonly tested port and BSU positions for two of the most frequently performed procedures—cholecystectomy, and left hemicolectomy combined with low anterior resection (Table 2)—are discussed in more detail.

**Table 2:** Surgical steps in cholecystectomy, and left hemicolectomy combined with low anterior resection

Procedure	Surgical steps
Cholecystectomy	Retract gall bladder Dissect anterior and posterior reflections of peritoneum Dissect Calot’s triangle and establish critical view of safety Ligate and divide cystic artery and cystic duct Dissect gallbladder from liver Remove gallbladder through balloon bag
Left hemicolectomy combined with low anterior resection	Locate duodenum and divide inferior mesenteric vein Perform medial to lateral mobilisation over kidney Enter lesser sac over pancreatic body and separate mesocolon from body and tail of pancreas Divide omental attachment to left half of transverse colon, enter lesser sac from above and continue around to fully mobilise splenic flexure Isolate and ligate inferior mesenteric artery approximately 1 cm distal to origin from the aorta Mobilise sigmoid colon medial to lateral anterior to Toldt’s fascia, preserving the left ureter, and then release lateral attachments Dissect mesorectum in total mesorectal excision plane starting right and right posterior, moving round to the left from underneath Dissect mesorectum on right side, left side and then anteriorly in rectovaginal septum or posteriorly to prostate, either anterior or posterior to Denonvilliers’ fascia

**Fig. 3:**
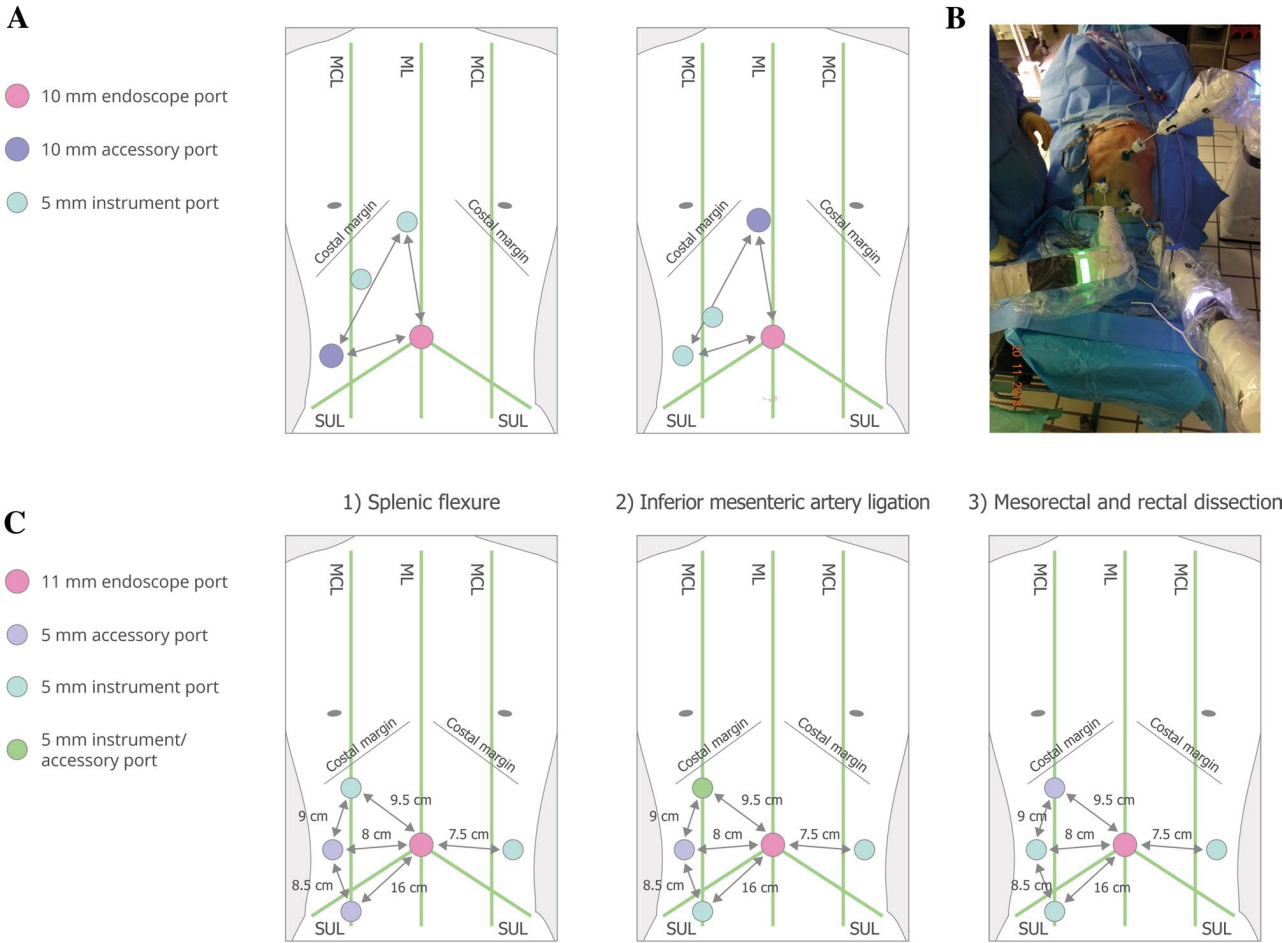
Common port positions tested in cadaver studies. **A** The two common triangular port configurations for cholecystectomy (13/17 procedures, 6/7 surgeons). For the second configuration, the lower instrument port was also used as an accessory port, and the other two instrument and accessory ports were each used as combined instrument/accessory ports. An endoscope angle of 0° was used in 1/13 procedures testing this triangular configuration; the endoscope angle was 30° down for the other 12/13. **B** Example set-up for cholecystectomy. **C** Port positions for left hemicolectomy combined with low anterior resection. The endoscope angle was 0° for this procedure. All port positions were based on surgeon preference. Umbilicus is where the ML crosses the SUL. Diagrams not drawn to scale. *MCL* midclavicular line, *ML* midline, *SUL* supine-umbilical line

**Fig. 4:**
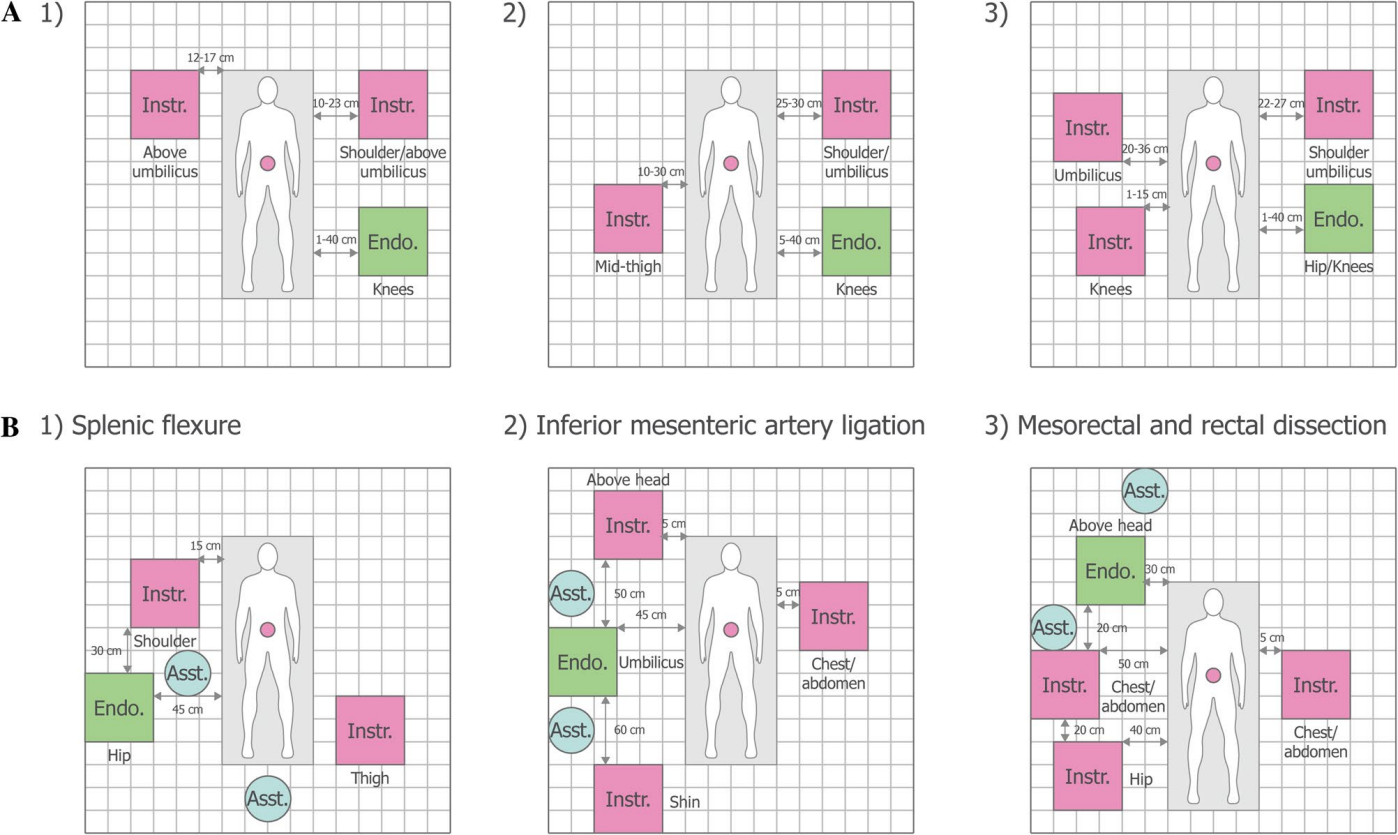
Common BSU positions tested in cadaver studies. **A** Common BSU positions for cholecystectomy: (1) 6/17 procedures, 4/7 surgeons; (2) 6/17 procedures, 4/7 surgeons; (3) 5/17 procedures, 3/7 surgeons. **B** BSU positions for the third combined left hemicolectomy and low anterior resection procedure. The position of assistant was not recorded for all procedures; the operating surgeon is located outside of the grid area at the surgeon console. The superimposed rectangle represents the surgical table with measurements detailing the distance between instrument bedside units and the surgical table or other bedside units. Diagrams not drawn to scale. The red dot indicates the umbilicus. *Asst* surgical assist, *BSU* bedside unit, *Endo* endoscope, *Instr* instrument

The cadaver position was supine for all cholecystectomy procedures. In the most common port configuration tested (13/17 procedures, 6/7 surgeons), ports were organised in a triangular configuration similar to port positions used by the surgeons for manual laparoscopic surgery (Fig. 3A and B). Additional port positions are detailed in Supplementary Fig. 3. Three BSU configurations were commonly used (Fig. 4A). All used one visualisation BSU located near the left knee; there was variation in the use of two versus three instrument BSUs across different locations.

For all combined left hemicolectomy and low anterior resection procedures, the cadaver procedure was supine; it was not possible to use the modified Lloyd-Davies position as only cadaver specimens (torsos) were available. In the first two procedures attempted, the port positions were found to be unsuitable: the first procedure could not be completed, and there was clashing between arms in the second procedure, although it was completed. Figures 3C and 4B show the port and corresponding BSU positions for the three major areas of dissection (splenic flexure, inferior mesenteric artery ligation, mesorectal and rectal dissection) in an alternative configuration used in the subsequent, successful third procedure. The port and BSU positions for the fourth and fifth procedures, both performed by the same lead surgeon, are presented in Supplementary Figs. 4 and 5. The port positions for these procedures were suitable, though low anterior resection could not be completed in one procedure due to the physical condition of the cadaver.

### Safety in live animals

Six cholecystectomy (non-recovery *n* = 2, recovery *n* = 4) and five small bowel enterotomy procedures (non-recovery *n* = 1, recovery *n* = 4) were performed in pigs. All procedures were successfully completed and there were no device-related intra-operative complications in any procedure; there were two non-device-related intra-operative complications recorded related to Veress needle insertion. Intra-operative blood loss was recorded as minimal (*n* = 1), negligible (*n* = 3) or none (*n* = 7). Clinical observations of the recovery pigs post-operatively revealed no signs of ill health or distress, and all recovery pigs gained weight post-surgery. Overall, recovery pigs remained in good health throughout the post-operative recovery period. There was standard post-operative swelling and/or scab formation at the surgical port sites; one pig required veterinary treatment (twice-daily with diluted chlorhexidine solution for two days) due to a thickened port site with overlying scab formation, although there was no evidence of infection.

**Fig. 5:**
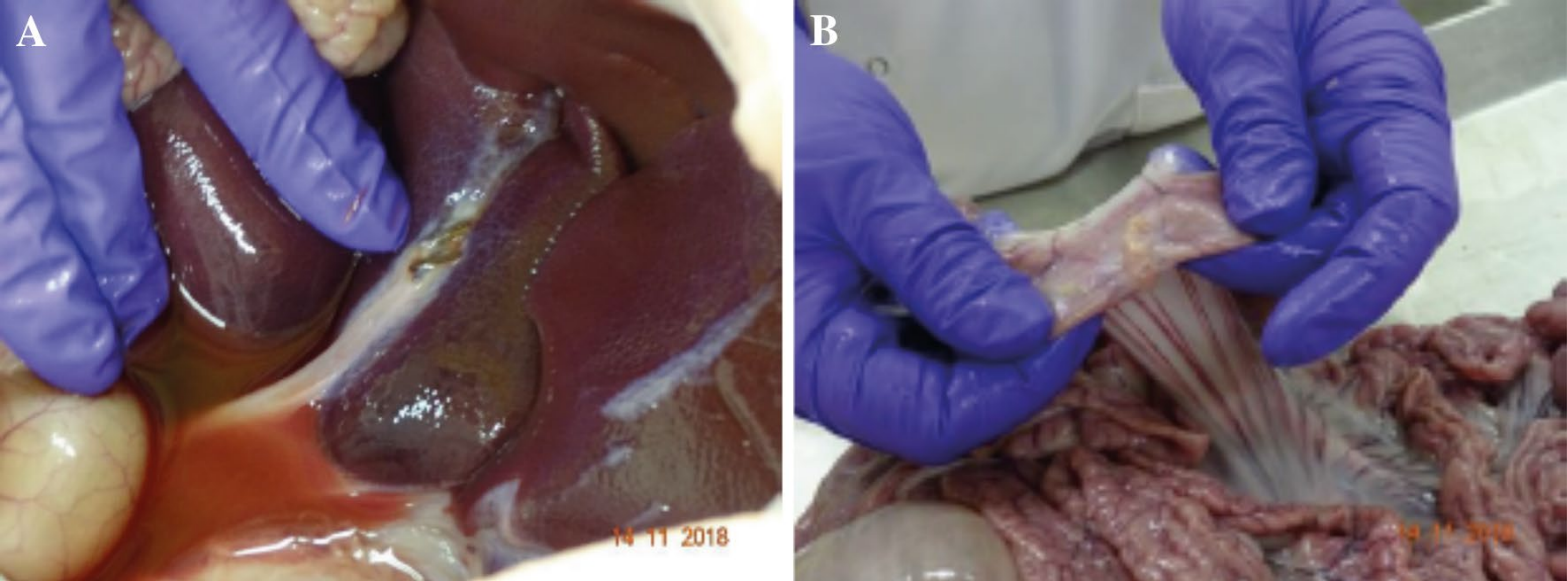
Necropsy findings from recovery pigs. **A** Necropsy of a cholecystectomy recovery pig showing macroscopic evidence of a healthy normal liver and good healing of the cystic duct and artery. **B** Necropsy of a small bowel enterotomy recovery pig showing no evidence of adhesions and bowel throughout looking healthy and normal

At necropsy, assessments showed that the majority of pigs had recovered well, with port sites healing, and surrounding organs appeared macroscopically healthy with no signs of injury, infection or inflammation (Fig. 5). A single loose clip was found in the abdominal cavity attached to the parietal peritoneum of one pig that underwent cholecystectomy.

## Discussion

Overall, the cadaver studies described demonstrate that the system can be used for MAS robotic surgery across a wide range of general and colorectal procedures. The system’s flexibility and portability enabled adequate surgical access and reach in the abdomen and pelvis, even in cadavers with a high BMI. The system’s user-led design (articulation of the arm, wristed instruments, ergonomic handgrip and console) enabled surgeons to successfully complete procedures requiring movement across the abdomen and within the confined space of the pelvis. The procedures described were performed in a manner reflecting how they would be performed in the clinical setting, from surgical set-up to the surgical steps performed. A preclinical assessment of the system for transanal total mesorectal excision in a cadaveric model has previously been published [16]; the studies presented here demonstrate the ability of the system to perform many other general and colorectal procedures. Moreover, the ability to perform cholecystectomy and small bowel enterotomy safely and effectively has been demonstrated in a live animal model, providing a good simulation of the system performance expected in live humans and demonstrating that the instruments could be used for the safe and effective manipulation of live tissue.

The port placement for robot-assisted laparoscopic procedures with other systems in routine use usually requires three or four ports for the robotic arms and one or two assistant ports. As such, enough space must be given between each port to permit freedom of movement for all arms while creating a working space for the bedside assistant. For example, a typical configuration for robot-assisted total mesorectal excision uses five or six ports, with all ports aimed towards and fanning out either side of the camera port, centred around the umbilicus [17]. Therefore, an operating surgeon is often unable to transpose their preferred manual laparoscopic port set-up to robot-assisted procedures. In contrast, findings from this study demonstrate that using this system, a variety of port placements provide adequate surgical access and reach; this flexibility enabled surgeons’ to effectively transfer their preferred laparoscopic port placements, when desired, for use with the robotic system. This may have the benefit of reducing the learning curve associated with robot surgery. The use of standard, disposable 5 mm ports for the operating arms further enhances the versatility of the system as users are not limited to the sites of dedicated robotic trocars. Overall, these are potential advantages of this system compared to existing robotic devices, which will be further explored in future clinical studies.

### Further development of versius

The system tested in these studies is not the final design. Incremental changes to instruments, hardware and software were made throughout these studies to improve the design of the robot and the surgical set-up for each type of procedure tested. Although the safe and effective use of the instruments was demonstrated by successful procedure completion in the porcine study, further studies will be performed to more quantitatively assess instrument functionality, particularly that of the electrosurgical instruments. In addition, procedures that have been performed a limited number of times in cadavers will be repeated to further optimise the use of the device for these surgeries. The aim is to ensure the robot and its use are perfected ahead of clinical studies in general and colorectal MAS surgery.

### Limitations

Human cadaver and porcine models are frequently used in surgical training, and each model has advantages and disadvantages in terms of its ability to test robotic surgical potential in live humans. Porcine models provide better handling of live tissues and a greater ability to dissect and identify planes than cadavers. However, cadavers provide much greater anatomical relevance and realism to live humans than pigs [18]. Testing in both human cadavers and pig models balances the bias introduced by each model; however, it is impossible to completely replicate the experience and performance of the robot for surgery in live humans. The subset of procedures performed in pigs was selected to provide a good simulation of system performance for general and colorectal surgery in live humans. The number of procedures performed was deemed suitable to generate sufficient evidence for the safety of the system whilst aligning with the 3Rs.

### Final conclusions

The studies presented here cover the comprehensive preclinical assessment of Versius for general and colorectal surgery in cadaveric and porcine models. Several types of general and colorectal surgeries were tested in cadavers, with the lead surgeons evaluating a range of port and BSU positions; all but one procedure was successful. Cholecystectomy and small bowel enterotomy were also performed safely and effectively in a live animal model. Overall, these results support the progression to assessment in clinical studies as per the IDEAL-D framework [14].

## Electronic supplementary material

Below is the link to the electronic supplementary material.

Supplementary file1 (DOCX 5057 kb)
