# Supplementary material for: Preclinical evaluation of the versius surgical system, a new robot-assisted surgical device for use in minimal access general and colorectal procedures
Source: Surg Endosc. 2020 May 13;35(5):2169–77. doi: 10.1007/s00464-020-07622-4 (PMC8057987; doi:10.1007/s00464-020-07622-4)
Supplement: Supplementary file 1 — Supplementary file1 (DOCX 5057 kb) [file 464_2020_7622_MOESM1_ESM.docx]

SUPPLEMENTARY FIGURES

Supplementary Figure 1. Schematic overview of the Versius Surgical System.

Adapted from Haig F, Chitty K, Medeiros T, et al. (in press). BSU: bedside unit.

**Supplementary Figure 2.** Record keeping of BSU positions. Outline of the grid template used to measure BSU location. To ensure reliable and standardised reporting of measurements, a grid of 20 cm x 20 cm squares was laid out on the OR floor such that the overall grid was 320 cm x 320 cm.

BSU: bedside unit; OR: operating room.

**Supplementary Figure 3.** The four additional port positions for cholecystectomy tested in cadaver studies. Each configuration was used in only one procedure; the same lead surgeon tested positions (B) and (C). The endoscope angle was 30^o^ down for all four procedures.

All port positions were based on surgeon preference. Umbilicus is where the ML crosses the SUL. Diagrams not drawn to scale. MCL: midclavicular line; ML: midline; SUL: supine-umbilical line.

**Supplementary Figure 4.** Port (A) and BSU positions (B) for the fourth combined left hemicolectomy and low anterior resection procedure. The endoscope angle was varied 0^o^ and 30^o^ down through the procedure.

This procedure could not be completed due to fecal impaction of the colon, prohibiting access to the rectum. All port positions were based on surgeon preference. In (A), umbilicus is where the ML crosses the SUL. Distances were unavailable for port configurations in (1) and (2). In (B), the position of assistant was not recorded in (3); the operating surgeon is located outside of the grid area at the surgeon console. The superimposed rectangle represents the surgical table with measurements detailing the distance between instrument bedside units and the surgical table or other bedside units. The red dot indicates the umbilicus. Diagrams not drawn to scale. BSU: bedside unit; MCL: midclavicular line; ML: midline; SUL: supine-umbilical line.

**Supplementary Figure 5.** Port (A) and BSU (B) positions for the fifth combined left hemicolectomy and low anterior resection procedure. The endoscope angle was varied between 30^o^ up and 30^o^ down through the procedure.


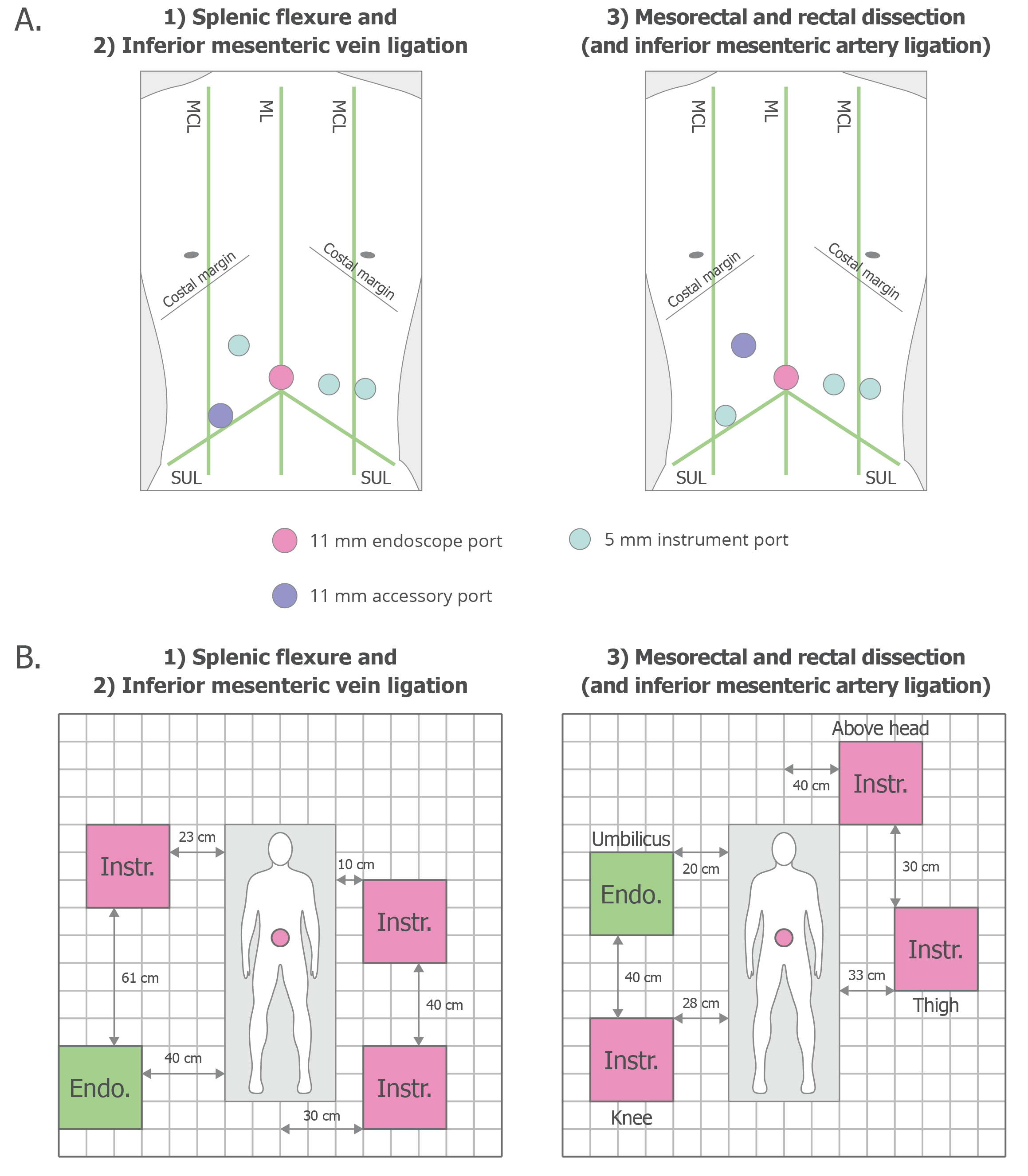


All port positions were based on surgeon preference. In (A), umbilicus is where the ML crosses the SUL. Distances were unavailable for port configurations. In (B), the position of assistant was not recorded; the operating surgeon is located outside of the grid area at the surgeon console. The superimposed rectangle represents the surgical table with measurements detailing the distance between instrument bedside units and the surgical table or other bedside units. The red dot indicates the umbilicus. Diagrams not drawn to scale. BSU: bedside unit; MCL: midclavicular line; ML: midline; SUL: supine-umbilical line.
